# Stanley Plotkin, MD, Discusses Scientific Progress and the Complexities of Public Health Vaccine Policy

**DOI:** 10.20411/pai.v10i2.891

**Published:** 2025-10-02

**Authors:** Neil S. Greenspan, Michael M. Lederman

**Affiliations:** 1 Case Western Reserve University, Cleveland, Ohio

**Keywords:** Vaccine Development, Vaccine Policy, mRNA, Mucosal Immunity, Public Health Policy

## Abstract

Stanley Plotkin, M.D., reflects on his professional journey, describing how formative educational experiences influenced his career. He discusses his contributions to vaccine development, notably for rubella and rotavirus, and shares insight on advancements in vaccine technology, including strengths and limitations of mRNA and DNA platforms. Dr. Plotkin emphasizes the importance of adapting public health recommendations as scientific understanding evolves, discusses the challenges of establishing mucosal immunity, and highlights the benefits of combining vaccines. He also offers thoughtful perspectives on natural versus vaccine-induced immunity, drawing upon his extensive expertise. Finally, he touches on his personal interest in learning to fly.

## NEIL S. GREENSPAN, MD, PHD

I am Neil Greenspan on behalf of *Pathogens and Immunity*, where I am one of the senior editors. I am joined by Michael Lederman, the editor-in-chief of *Pathogens and Immunity*. Today we have the good fortune to host a conversation with Stanley A. Plotkin, M.D., who received his under-graduate degree from New York University in 1952. He then received his M.D. in 1956 from the State University of New York Downstate Medical School in Brooklyn and completed his internship at what is now MetroHealth Medical Center in Cleveland, Ohio, which is an affiliate of our home institution, Case Western Reserve University.

Dr. Plotkin is a highly accomplished developer of and authority on vaccines, especially for viral pathogens. He played direct and pivotal roles in developing the rubella and rotavirus vaccines currently in clinical use and has consulted on or contributed to many more.

Dr. Plotkin has held both academic and corporate positions involving vaccine research and development. In addition, he is the founding editor of the definitive textbook on vaccines, Plotkin’s Vaccines [1], that is now in its 8th edition, and he has received many honors for his work on vaccines, including the French Legion of Honor in 2001, the Albert B. Sabin Gold Medal in 2002, the Maxwell Finland Award for Scientific Achievement in 2009, and the Hamdan Medical Research Excellence award in 2014.

Welcome Stanley.

## MICHAEL M. LEDERMAN, MD

Dr. Plotkin. Thank you so much for joining us here. My first question is, what are the influences that drove you into science and medicine?

## STANLEY PLOTKIN, MD

When I was 15, I read two (important) books. One was Microbe Hunters by Paul de Kruif [2] and the other was Arrowsmith, by Sinclair Lewis [3]. I know it sounds exaggerated, but they convinced me that I wanted to go into biological science and to work on vaccinations. So, I applied to and graduated from the Bronx High School of Science, which still exists, and which is a magnificent institution in New York City. I then went to New York University, which at the time had a campus on the western side of the Bronx, overlooking the river and Manhattan. I then went to medical school at what is now called Downstate Medical Center in Brooklyn, NY.

## NSG

I wanted to ask you, since I read Arrowsmith in college, were there any particular aspects of the main character’s wrestling with research versus clinical medicine that particularly affected you?

## SP

What struck me in Arrowsmith was the evolution of his thinking and his direction. As you may recall, the book is filled with tragedy, but he ultimately ends up at what was the (fictional equivalent of the) Rockefeller Institute, and he becomes satisfied with his work. It was not that I was emulating his career, but his desires aligned with my growing desires. And so, it was definitely something that stimulated me to go in that direction.

## MML

So, you’ve lived in both worlds, both the clinical and the research world. How do you balance the tension between clinical responsibility and scientific enterprise?

## SP

Well, that’s, of course, a very good question, and the answer is, by working 20 hours a day.

I interned at Cleveland Metropolitan Hospital. I went there, in part, because Fred Robbins (who had worked with John Enders) was the head of pediatrics at Cleveland Metropolitan. As I was finishing my internship, it was a time when military service was obligatory. I was going to go to the Air Force after my internship when one of my fellow interns told me about the Epidemic Intelligence Service at the Centers for Disease Control (CDC). I applied for entrance and was accepted, so I never went to the Air Force. I learned about epidemiology.

At the end of the CDC training, there was a list of public health service assignments. One of them was at the Wistar Institute in Philadelphia. It was in an anthrax laboratory. I had no particular interest in anthrax, but I knew that Hilary Koprowski had taken over at the Wistar Institute, and my reasoning was that if I went to the institute, I could get into his laboratory. So, I learned about anthrax.

By chance, an epidemic of inhalation anthrax occurred in Massachusetts, so I went up and worked on that and then published on anthrax. And then, I walked into Koprowski’s office and asked him if I could get into his lab, and he agreed. So, I started working simultaneously on virology and bacteriology. I became involved in his oral polio vaccine project, went to the Congo to test the vaccine. As I said before, chance is everything in life, and I was very lucky to have those opportunities and to take advantage of them.

## NSG

I’d like to transition now a little bit away from your personal history and into what you think about vaccines and medical problems as they are evolving. Do you have any conclusions or lessons that you take from the COVID-19 pandemic?

## SP

What the public doesn’t recognize, but which is fact, is that COVID was an entirely new disease with new biological qualities. We started of course with ignorance about the properties of the virus and the disease, and science learns by experience, by experiments, etc.

The recommendations that were made at the beginning of the outbreak were in line with what was known at that time. The virus was quite virulent in its first manifestations. But of course, with time, the virus evolves. Mutations allow it to infect more people but kill less. And if you want to put it this way, the aim of the virus is not to kill people, but to multiply and spread. It adapts. So, the virus changed, and our public health recommendations changed.

Now, the problem is that the public does not understand science; scientific education in this country is terrible, by and large. The public does not understand that things change and (as a result) recommendations change. So now you have a situation where people are complaining that the original recommendations were wrong; they were not. But now we have different recommendations, and I understand that.

I agree, for example, that young males should not be vaccinated against COVID-19 anymore, because if they get infected, their disease will be mild. And they probably have had prior infection anyway. And the reaction rate (to the vaccine) is significant.

On the other hand, pediatric COVID-19 disease, particularly under the age of 3, can be quite serious. Therefore, I do think introductory vaccination in children, which gives them some resistance against the virus later on in life, is a good idea. Now, what I’m getting at is that public health recommendations should be made based on the current facts, but the public doesn’t understand that our understanding changes.

## NSG

How do you assess the performance of the mRNA vaccines for SARS-CoV-2? And do you think there are any aspects of this vaccine platform that underperform relative to the older protein-based subunit vaccines?

## SP

Well, the induction of T cell responses wasn’t optimal, and the dosage was pretty high. And we’ve learned how to change the technology, for example, using a self-amplifying mRNA to reduce the amount of RNA going into the patient. So, what I would say is that mRNA was a great emergency response. Science can now allow for reduction of (vaccine-related) reactions. And there is a need for improvements in T cell responses induced by mRNA vaccines. And, actually, I have written a paper contending that polytheism is preferable to monotheism [4], with the idea being that everything should not go into the mRNA basket. We have other technologies that provide good immunity, and in some cases, better cellular immunity.

## MML

Well, with those things in mind, do you think that there are any current older platform vaccines that could or should be replaced using an mRNA platform?

## SP

So, we have the live attenuated vaccines. They generally give both B and T cell responses and prolonged protection. So why would we replace those in favor of mRNA? And there are other situations, for example, Chikungunya, where you could make an mRNA vaccine, but what would be the advantage? You’ve got the live vaccine and the killed vaccine now, which are very effective. So why would you switch to mRNA?

## MML

What about, perhaps, as an option for the rare, immunocompromised patient for whom an attenuated vaccine has the risk of dissemination?, Would that be a space for either killed or protein-based, or mRNA-based vaccines?

## SP

I suppose that would be a reasonable possibility. But you would want to know that the immune responses were sufficient with the replacement vaccines, and particularly the T cell responses.

The other day, I had a question about mRNA vaccines for immunosuppressed people; the idea being that, for example, in somebody who couldn’t produce antibodies very easily, but still has T cell activity, an mRNA vaccine would not be ideal. You would want something that would stimulate a good T-cell response to provide whatever possible immunity that T cells could give to that immunosuppressed individual. I think one has to evaluate each situation and decide what is the optimum.

## MML

Why do you think the T-cell response to mRNA vaccines is suboptimal? What underlies that failure?

## SP

I think the immunogen goes to the bone marrow. The B cell response is pretty good, but apparently, it doesn’t get into lymph nodes where T cell stimulation can occur. But of course, it’s not black and white. There are T cell responses to RNA vaccines, but they’re not great, and they don’t last.

## NSG

You may recall that, during the pandemic, a paper in the *New England Journal* explored the notion that you could now just sequence a virus and generate an mRNA vaccine in 100 days and start immunizing people [5]. I was taken aback by that, because I think it was underappreciating the 20 years or more of research that underlies the successes of the mRNA approaches in the case of COVID.

What do you think about this idea that was floated in this article, that you could produce new vaccines in 100 days from the time of identification?

## SP

Well, you know, I’ve been very involved from the beginning in CEPI, the Coalition for Epidemic Preparedness Innovations. It is an organization that I and a couple of others proposed about 10 years ago to develop emergency vaccines and vaccines that had no commercial interest [6].

I think mRNA vaccines are excellent for rapid responses. When a new disease emerges and a quick vaccine is needed, such as was the case with COVID, mRNA is a great technology. But in the long term, you want a vaccine that is well tolerated and that provides long-lasting immunity. My view — which is essentially reflected in CEPI — is that you have an emergency response, then over time, focus on developing a vaccine that offers the most extensive and lasting immunity. And so, I think the idea of mRNA — for a new disease is a great idea, because it’s quick. But you also have to think long-term. What’s the best way of controlling the disease after an emergency response?

## NSG

I guess implicit in the question for me is the notion that in the case of SARS-CoV-2, it was pretty obvious that a single protein was going to be protective, based on its enormous similarities with the original SARS virus from earlier in the 2000s. I think with a truly new pathogen, even if it’s part of a known family of viruses, it may not be as clear what antigen is worth mass-producing immunity against. There are also safety issues, which I don’t think can be appropriately evaluated in the absence of pathogenesis work, and (some of) the people who were pushing the 100-day idea, were suggesting that there should be no live animal work with new viruses. There may be cases where that would be too dangerous. But it seems to me it’s kind of dangerous to produce a vaccine that you disseminate widely and immunize millions of people with before you know what the range of possibilities are with a given virus, based on your own law, which was that any virus can do anything.

## SP

I understand your point, but in 100 days, you can do animal studies. So you can get some safety data in animals. And you can still do phase 1 studies in a small number of people. The point I’m trying to make is that there should be different approaches to emergency situations and to long-term control. You do what you can as quickly as you can to provide an emergency response. But meanwhile, you’re studying the biology and calculating what, in the long term, is the best vaccine approach to a particular disease.

I guess an example would be the monkeypox situation in Africa. In retrospect, I think an mRNA vaccine definitely would not have been the best choice, but it might have been a way to save lives initially. But, clearly, in the long run, a monkeypox vaccine is not going to be an mRNA vaccine. But it might have been a quick way of getting many people protected in a short time. The thing what’s remarkable to me is that when I started, the only options were basically attenuation or inactivation. But now you have multiple technologies, which is great.

## MML

We’ve all seen persons who’ve had multiple instances of COVID-19 over time. Is this primarily (due to) viral evolution? Is it a non-durable immune response both to vaccine and perhaps to the natural infection. What should we be thinking about in terms of herd immunity to COVID-19?

## SP

People do get reinfected because the disease is primarily a mucosal infection, and we don’t do very well in protecting the mucosa. This is probably the biggest area of ignorance in vaccinology: how to induce mucosal responses.

I’m very intrigued by the approaches being studied that combine a poxvirus method with a method to stimulate antibodies using, say, mRNA or other technologies. In other words, aim to activate both cellular immunity and antibody responses because that’s more natural, so to speak. The combination provides the best natural immunity, but we still need to do a lot more work on the mucosal part of it. We know that live viruses given on the mucosa do stimulate mucosal responses, but we don’t have safe live viruses for every disease.

The issue is how to safely induce mucosal responses. And at the moment, I would say that our knowledge of that is very primitive. We just don’t know the best way to do that, aside from, as I said, a live agent that’s multiplying locally on the mucosa. So, this, to me, is one of the biggest areas of ignorance we have in vaccinology.

## MML

You mentioned briefly the role of poxvirus. So, what do you think about a DNA platform for immunization? Do you think we can generate a good DNA platform for immunization?

## SP

We have a DNA platform. The problem is that it requires an apparatus to administer it intradermally. The people at Wistar Institute have long worked on this. However, it hasn’t gone anywhere for practical application, because the idea of using an apparatus to give intradermal injections is simply not popular. I’m still consulting with that group and trying to develop better ways to make use of the DNA platform, which has a lot of appeal, and it does produce good cellular responses, but so far we haven’t figured out a way to eliminate the need for an apparatus to administer the injection.

## NSG

You mentioned earlier that you worked on poliovirus when you were at Wistar as part of Hilary Koprowski’s program. I’m just wondering what you think about the trade-offs in general between live attenuated and subunit or other non-replicating vaccines. The most prominent example of the risks of live attenuated vaccines is polio. And in the context that Michael previously alluded to of immunocompromised patients where you can actually get polio spread. What are your most recent thoughts about, for example, the poliovirus situation, where there is still an argument about stopping Sabin (live attenuated) vaccinations.

## SP

Let me put it this way. The oral polio vaccine was tremendously valuable in stopping the outbreaks and the spread of polio viruses. From the public health point of view, it was a tremendous success. However, in the United States, as time went on, wild-type polio disappeared, and what was left was the 10-plus cases a year of vaccine-derived polio, which became unacceptable. And so, there was a switch to inactivated polio vaccine and the eradication of polio from the United States and the Western hemisphere. Now you have the situation where wild-type polio persists in a small part of the world. Lots of people are receiving attenuated oral polio vaccine, and more paralysis is being caused by the oral vaccine than by the wild-type virus. So, it’s time to switch our strategy. At this point, what I and others advocate is that the inactivated polio vaccine should be used as widely as possible, preferably in combination with other pediatric vaccines, and that the use of the oral live attenuated polio vaccine should be discontinued as much as possible because it’s causing more disease than the wild-type virus. That’s an example of how recommendations change with the public health situation. If every child received inactivated polio vaccine, you could even give oral polio vaccine later in life because they would be protected against paralysis, so you could use both on the condition that the child had the induction of antibodies before being exposed to the to the oral polio vaccine.

## NSG

My next question has to do with a recent paper published in February of this year, which you may not have seen, in which a group of collaborators from Stanford and Pasteur, Yasmine Belkaid and Michael Fischbach and others, came up with the idea of using a vector that would infect commensals in the skin to immunize against true pathogens that could cause skin infections [7]. I’m not expecting you to critique the paper. I just want to know what you think of their general idea, which was to use a cleverly designed vector to get sequences into commensal skin microbiota. The next step is to use that to immunize against potential skin pathogens in a context where risk of infection with that pathogen is significant. I just wanted to get your reaction to that concept.

## SP

Well, it sounds like an intriguing idea. So, it would be an organism that normally lives on the skin, which is not a pathogen, but which is producing some protein that protects against another agent?

## NSG

Yes. I don’t remember the exact nature of the vector, but it’s sort of like an mRNA vaccine in that you’re using the host to actually biosynthesize the relevant antigens. But instead of using host cells, you’re using commensal microbes. People have also talked about that in the gut, but I just thought it was an interesting wrinkle that I had not seen before.

You’ve already indicated your understanding of the importance of eliciting strong cell-mediated responses. I did my graduate work at Wistar with Peter Doherty, and we were focused on the CD8+ T cell response, in particular, to influenza. So, although it was clear that CD8+ T cells couldn’t prevent infection, they were quite important in resolving infection. That was one of the main lessons of that work.

## SP

Immunity is complex. This is may be irrelevant, but I was approached recently by a company that has a system of measuring antibodies to vaccine antigens. Their idea was that people would be tested for antibodies, and if they were lacking an antibody, that would be an indication for re-immunization. The problem was that one of the antibodies they were testing was a human papillomavirus antibody. I had to point out that antibody levels fade, but we know from experience, because of the long incubation period of the virus, that there’s a memory response years after the initial vaccination. So, you don’t need revaccination because the memory response is there, and it protects you against the disease. Obviously, they were not happy with this. But one has to think about the immune responses and what correlates with protection rather than simply having a number.

## MML

Talking about multiplicities in clinical practice, we often immunize with more than one immunogen. Is there a limit to how many we can apply to a person at any one time, and if so, what would that limitation be?

## SP

Well, of course, that’s a good question, and the only answer I can give is, whatever the limit is, we haven’t reached it. I can’t give you a number, but people are now receiving really large numbers of antigens, and so far, we have not seen a limitation.

## NSG

And that’s primarily in children right?

## SP

Yes.

## MML

What about in the military? Aren’t they immunized with multiple immunogens at the same time? So maybe the issue is primarily one of tolerance (of vaccine side effects). How much of a sore arm can you tolerate?

## SP

Well, that’s true. But similarly, there is now an important need to combine vaccines because of the resistance (in a behavioral sense) people have to multiple injections. I have witnessed and heard and responded to people complaining about the number of injections that their child is getting. And I understand that. Companies are working hard on trying to combine as many antigens as possible. And as we were just saying, so far, we haven’t reached a limitation for that. But it becomes very important from the public health point of view. People start objecting to the number of injections; obviously, that reduces the coverage. So, we need combination vaccines.

## MML

Before I turn this over to Neil, is there evidence for cross-vaccine enhancement such that the concurrent administration of an unrelated vaccine enhances the immune response to the other vaccine? They may go to lymphoid tissue, they may go to marrow. Is there the sense that giving more than one enhances the response to the other?

## SP

Offhand, I can’t think of an example of that. But I suppose the answer to that could become evident in studies comparing multiple antigens with single antigens. I’m not aware of any data suggesting that, but it’s a good question. It could possibly be answered by companies developing combination antigens and comparing them.

## NSG

I don’t recall ever seeing a paper about that topic, but that doesn’t mean it doesn’t exist. I have a quick comment on that. Years ago, and for many years, I had what I presumed was a tiny papillomavirus-related lesion on the exterior surface of my left index finger. It was less than a millimeter across. After I got the shingles vaccine, it completely disappeared. I don’t know if it means anything, but it was truly interesting that it was clearly (temporally) related to the shingles vaccine. I don’t know what that says about cross-reactivities or innate immunity, or what. It may be irrelevant, but it’s a potentially interesting anecdotal observation.

Do you have any general comments about infection-induced versus vaccine-induced immunity to the pathogens for which we typically immunize? I know that there was a political valence to that for a while. I’m not interested in that so much as just your thoughts about whether there’s any reliable difference, or whether you think it’s very context dependent.

## SP

With a vaccine, usually you are selecting the antigens that you think are most protective. Obviously, with an agent, you’re getting all of the antigens. So, I suppose there might be some argument that getting the disease gives you better protection than getting the vaccine. But obviously, the cost of that is the disease, and clearly, what we do in the lab is to try to find the antigen that most corresponds with protection without using the other antigens, which may also induce (undesirable) reactions. So, it’s a trade-off. However, I believe the practical results clearly demonstrate that protection can be achieved with a single or possibly two or three antigens, without inducing reactions to other parts of the agent. So, maybe, theoretically, disease-induced protection is better than vaccine-induced protection. But then, of course, you’re paying a price.

## NSG

One thought I had is that in addition to the points you’ve made, there’s also the fact that pathogens are often selected to be able to subvert various immune responses. So, while it’s true that you’re obviously getting the more natural assembly of antigens with pathogen infection, you’re also subject, not just to disease, but to other mechanisms, which may not be visible, that are undermining your immune response.

## MML

HIV is a classic example. You’ve got a pathogen that potentiates its pathogenicity by impairing or deleting immune cells that it affects directly and indirectly. When we immunize, we typically use an adjuvant, so should all vaccines contain an adjuvant, and if so, do you have a favorite — or a favorite in one setting versus another?

## SP

Well, the last question is easy to answer. No, I don’t have a favorite. I think one of the positive things that’s happened over the years is the development of new adjuvants beyond aluminum. Now we have multiple choices, which is valuable.

I did work in the past with MF59, which I thought was really, really good and which seemed to be less reactogenic than aluminum. So, I’m very much in favor of developing a wide range of adjuvants, because I think there are differences. And of course, differences in reactogenicity. So, rather than being against adjuvants as some people are, I think the more the better. And there’s now quite a long list of reagents that can be used.

## NSG

Can you tell us why the standard influenza vaccine did not have an adjuvant? I’m just curious if you know what the consideration was.

## SP

I think it was to avoid or minimize any reactions that might occur.

We could definitely have better influenza vaccines. But for reasons which are not entirely clear to me, the companies have been reluctant to really get into the depth of influenza vaccinology.

## NSG

Maybe it’s because it’s an annually recommended vaccine, and if it had a lot of reactogenicity, it would probably have even lower uptake than it currently does. I don’t know, that’s speculation.

## SP

It could be. But, based on the science, we could certainly have better influenza vaccines.

## NSG

To wrap up, why did you want to learn to fly, and why do you find it a source of gratification?

## SP

Well, as I mentioned earlier, I was originally going to join the Air Force, one of the reasons being that I wanted to learn to fly, but then, of course, that plan got sidelined. So, when I was in my 70s, I decided to learn how to fly. There’s an airport about 5 miles from where I sit. I took lessons and eventually bought a single-engine plane. Why did I do that? Because of the feeling of lifting off the earth. The moment of leaving the ground is exquisite. To imitate a bird and to be able to see the earth from above is really different. Of course, in the commercial plane, you just don’t get that experience anymore. It’s a terrible experience now. But in a private plane, you can really see things. It’s not just a practical thing, it’s the sensation of being in the air and lifting off the ground, which is just something that I wanted to experience, and I loved it. But I gave it up because you have to have quick reactions. And I realized that I couldn’t do that anymore. And so, it was safer to give it up.

## NSG

I’m just struck by the fact that not only are the pathogens you study airborne sometimes, but so are you.

## MML

After that beautiful answer, I even hesitate to ask the bonus question. I understand you grew up in New York, and I’m guessing that you grew up in the Bronx. So, my question is, are you still a Yankees fan, and if so, why?

## SP

Well, I guess you could say, formally speaking, I’m still a Yankees fan, although you know, I’ve learned over the years that these guys are not New Yorkers. They come from all over the world, that they are essentially getting a lot of money for a job, and so I’m not the fan that I used to be. But when I was a kid, I was definitely a Yankees fan.

## MML

It’s good to hear that your allegiance has changed both in magnitude and phenotype.

So, Dr. Plotkin. Thank you so much for speaking with us. It was truly wonderful.

I think I can speak for Neil and the other editors of the Journal, that we’re so pleased that you were able to spend some time with us.

## NSG

It’s been a real delight and a privilege to have a chance to speak with you.

## SP

Thank you.

## SUPPLEMENTARY DATA

Supplementary materials are available at the *Pathogens and Immunity* website. Supplementary data may be provided by the authors to benefit the reader. Supplementary data are not copyedited and are the sole responsibility of the authors. Questions or comments related to supplementary materials should be addressed to the corresponding author.
